# African countries established COVID-19 testing in one month: Here’s how they did it

**DOI:** 10.4102/ajlm.v10i1.1457

**Published:** 2021-12-15

**Authors:** Timothy Amukele, Ryland N. Spence

**Affiliations:** 1 ICON Clinical Laboratories, ICON Clinical Research PLC, Farmingdale, New York, United States; 2 Department of Pathology, Johns Hopkins School of Medicine, Johns Hopkins Bayview Medical Center, Baltimore, Maryland, United States

**Keywords:** testing capacity, COVID-19, pandemic, coronavirus, Africa, laboratory

## Abstract

**Background:**

As a novel and deadly acute respiratory syndrome, which later became known as coronavirus disease 2019 (COVID-19), spread beyond China in late January 2020, there were no laboratories in Africa that could test for the disease. However, in early March, just over a month later, 42 African countries had developed the expertise and resources to perform COVID-19 testing. Our goal was to document this public health success story, learn from it, and use it to inform future public health action.

**Intervention:**

Three groups were primarily responsible for establishing COVID-19 testing capacity in Africa. The first group comprised early test manufacturers who reacted with incredible speed and ingenuity early in the pandemic, such as the German company TIB MolBiol that developed a molecular test for COVID-19 *before* the SARS-CoV-2 genome sequence was available. The second group included private and public donors such as the Jack Ma Foundation, and the last were the coordinators of the rollout, such as the World Health Organization and the Africa Centres for Disease Control and Prevention (CDC).

**Lessons learnt:**

The first lesson was that speed is critical, especially during a crisis. It was also demonstrated that being a predictable and transparent trusted institution opens doors and improves effectiveness. Africa CDC, which was only three years old, was able to secure significant resources from external partners and rapidly build substantial testing capacity within Africa because it is a trusted institution.

**Recommendations:**

Low- and middle-income countries must build local trusted institutions to better prepare for public health challenges.

## Background

When Côte d’Ivoire had its first suspected coronavirus disease 2019 (COVID-19) case in late January 2020, the nearest laboratory capable of performing COVID-19 testing was in Paris, France.^1^ Yet just over a month later, by early March, 42 sub-Saharan African countries had acquired the instruments, reagents and know-how to perform laboratory diagnostic testing for COVID-19.^2^ Broadly speaking, ongoing COVID-19 infection can be diagnosed using two types of tests: antigen-based and nucleic acid-based tests. Antigen-based tests detect the virus’s coronal spike proteins from which the virus derives its name. Nucleic acid-based tests (also called molecular or polymerase chain reaction [PCR] tests) work by directly detecting severe acute respiratory syndrome coronavirus 2 (SARS-CoV-2) RNA. These molecular tests are more specific and sensitive than antigen-based tests but are 3–10 times more expensive and require more complex instrumentation.^3^ Nevertheless, in early 2020, the overwhelming majority of COVID-19 tests available globally were molecular. In addition, although sub-Saharan Africa had less than 1% of the molecular testing capacity of that in the United States, thanks to HIV control efforts, every country had at least one location that could perform molecular testing once the SARS-CoV-2 molecular reagents were available.^4^ This article tells the story of how 42 African countries were able to acquire COVID-19 PCR testing capacity in two months in early 2020.

## Description of the intervention

### Ethical considerations

This study followed all ethical standards for research without direct contact with human or animal subjects.

### Data collection

We started by independently corroborating the ‘42 countries’ figure announced by the director of the Africa Centres for Disease Control and Prevention (CDC) in his interview on 11 March 2020.^2^ Specifically, we searched the internet and social media platforms such as Twitter for announcements by individual countries that they could now test for COVID-19. Next, we used Google searches to identify relevant sources (including those in the grey literature and popular press) that detailed *how* COVID-19 testing capacity was established in Africa in the first two months of 2020.

### Data analysis

**FIGURE 1:**
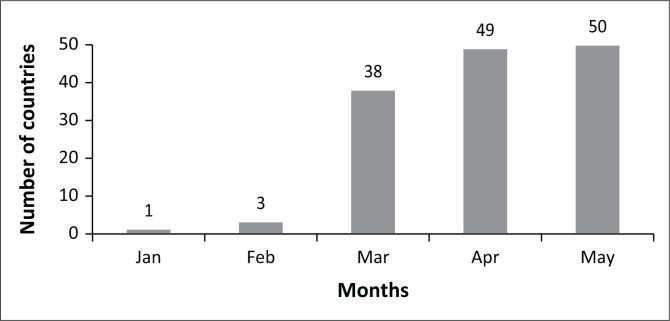
The total number of sub-Saharan African countries reporting coronavirus testing results or laboratory testing capacity, January 2020 – May 2020. Additional country-level data is available on the COVID-19 testing dashboard of Pathologists Overseas (https://www.pathologistsoverseas.com/).

Raw data were collected as described above, entered into Excel (Microsoft Corporation, Redmond, Washington, United States), and presented in this study without any transformation. No additional statistical analysis was performed. The figure displaying these data (Figure 1) was also created using Excel (Microsoft Corporation, Redmond, Washington, United States).

## Lessons learnt

As of March 2020, the total number of countries reporting coronavirus testing results or laboratory testing capacity in sub-Saharan Africa was 42 (Figure 1). This public health success story involved three principal actors: early manufacturers of COVID-19 tests, private and government donors, and coordinating bodies, including the World Health Organization (WHO) and Africa CDC, that coordinated efforts within and across the African countries. The first lesson learnt from this experience was that speed is critical during a crisis, as with the much-criticised motto of Facebook’s founder: ‘move fast and break things’. While this may not be good advice for every situation, it was the approach successfully adopted by all the actors in this story. The activities of these major players are discussed in greater detail below.

### COVID-19 test manufacturers

Test manufacturers reacted with incredible speed and ingenuity in the early days of the pandemic. To illustrate, the announcement of the isolation of the SARS-CoV-2 virus on 07 January 2020 and the determination of its genome sequence on 10 January 2020, occurred less than a week after China first shared information about a new and deadly flu-like respiratory illness with the WHO and other countries on 03 January 2020.^5,6^ While the speed of virus isolation and genome sequencing was remarkable in itself, something even more prodigious was accomplished by an early COVID-19 test developer. The German company TIB MolBiol (TIB MOLBIOL Syntheselabor GmbH, Berlin, Germany), in collaboration with the Charite Hospital in Berlin, announced a molecular test for COVID-19 on 10 January, the same day the SARS-CoV-2 genome sequence became available.^7,8^ They accomplished this feat by creating their molecular SARS-CoV-2 test based on the sequences of other coronaviruses.^9^ This is akin to sewing custom clothing for a stranger based on pictures of their parents and siblings. TIB MolBiol made several versions of this blind test and vetted them using actual COVID-19 patient samples before selecting the one that worked best. It was a gamble, but it paid off. The protocol for the best version was published by the WHO on 17 January 2020, and TIB MolBiol had sold four million tests by early March 2020.^10,11^

### Coordinating bodies

Many of the aforementioned TIB MolBiol tests were purchased by the WHO with funding from the Bill and Melinda Gates Foundation and sent to countries around the world to support the scale-up of the novel coronavirus diagnostic efforts.^12^ In particular, reagent kits were shipped to more than 20 countries in the African region by the WHO before 31 January 2020.^13^ This was to expand diagnostic capacity beyond the two referral laboratories in Senegal and South Africa that had it at the time. Of note, the WHO had a much more expansive role in responding to the COVID-19 pandemic.^13^ For example, on 31 January 2020, the WHO identified 13 top-priority countries that had either direct links with or a high volume of travel to China (Algeria, Angola, Côte d’Ivoire, the Democratic Republic of the Congo, Ethiopia, Ghana, Kenya, Mauritius, Nigeria, South Africa, Tanzania, Uganda and Zambia), and subsequent active screening was established in a majority of the airports in these countries.^13^ However, these initiatives that are not directly about the establishment of COVID-19 diagnostic capacity in Africa in the first month of 2020 will not be covered in this focused report.

**FIGURE 2:**
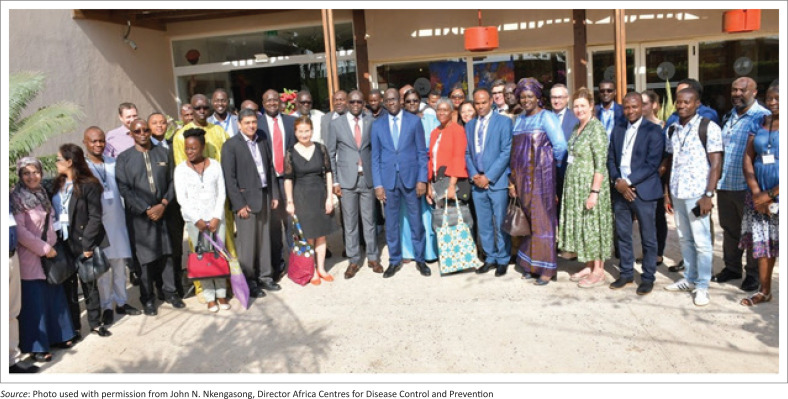
First training on laboratory diagnosis of COVID-19 in Dakar, Senegal, 06–08 February 2020.

In Africa, the WHO-sourced kits were primarily distributed by Africa CDC, a technical institution of the African Union that was established in 2016 but officially launched in January 2017.^14^ Like other actors in this story, Africa CDC acted swiftly. On 22 January 2020, five days after the publication of the TIB MolBiol protocol on the WHO website, Africa CDC announced that it was working with member states to identify laboratories that were capable of receiving and testing specimens.^10,15^ On 05 February 2020, they created the Africa task force for coronavirus preparedness and response,^16^ a multi-country multi-agency group designed to collaborate, communicate and coordinate efforts in response to the coronavirus pandemic. A day later and exactly a week after announcing that they were identifying laboratories in member countries, Africa CDC, in collaboration with the Pasteur Institute in Dakar, organised a workshop and training on COVID-19 diagnosis for medical teams from 16 African countries, where each trainee received a kit that could run 100 tests (Figure 2).^17^ The countries were Côte d’Ivoire, Cameroon, the Democratic Republic of the Congo, Egypt, Ethiopia, the Gambia, Gabon, Ghana, Kenya, Nigeria, Morocco, Senegal, South Africa, Tunisia, Uganda, and Zambia.

**FIGURE 3:**
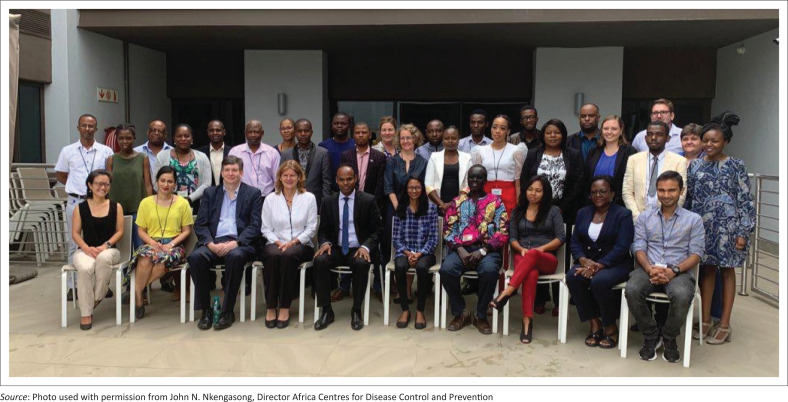
Second training on laboratory diagnosis of COVID-19, South Africa, 21 February 2020.

In partnership with the National Institute for Communicable Disease, a second training on laboratory diagnosis of COVID-19 was organised by Africa CDC in South Africa on 20–22 February 2020 (Figure 3). Each of the 12 countries that took part in the training received kits for the testing of 192 specimens, while Egypt was provided with 700 additional kits, and Nigeria and Rwanda each received 1000 additional kits.^18^

As many of these groups that were trained lacked the equipment to perform the testing in their home countries, the procurement and placement of equipment and biosafety protective gear were also addressed by the WHO and Africa CDC. In addition, as stated in a media briefing on 04 February 2020, Africa CDC planned to set up a referral system where the 16 laboratories that would be set up to perform COVID-19 testing could receive samples and support other countries who were unable to test.^19^ It is not clear if this referral system was ever established but it likely was not needed for long because by 11 March 2020, John Nkengasong, the director of Africa CDC, had announced in an interview that there were 42 African countries with COVID-19 testing capacity at the central level.^2^ Like the WHO, the efforts of Africa CDC transcended laboratory testing and included significant efforts in five other high-priority areas for coronavirus control, namely surveillance, infection prevention and control, clinical care, risk communication, and the distribution of donations by other groups and individuals.^20^

Pre-existing disease surveillance networks also helped in the response to COVID-19 in Africa. For example, the WHO has 27 WHO Collaborating Centers (defined as ‘institutions such as research institutes, parts of universities or academies, which are designated by the Director-General to carry out activities in support of the Organization’s programmes’) in 14 African countries.^21^ Other laboratory networks in Africa include the Regional Integrated Surveillance Laboratory Network, which is run by Africa CDC and based in Zambia, Kenya, Gabon, Nigeria, and Senegal; the West African Network of Biomedical Analysis Laboratories, which is a network of laboratories in francophone West Africa supported by the Mérieux Foundation^22,23^; and many other disease-based laboratory networks. These laboratories were already in close contact and sharing surveillance and other information with the WHO or CDC before COVID-19. Thus, they were logical sites for situating COVID-19 testing once the kits became available.

### Private and government donors

One of the most significant donations to help counter the impact of the coronavirus in Africa was that made by the Chinese billionaire and founder of Alibaba, Jack Ma.^24^ Jack Ma’s massive operation had, by September 2020, shipped over 200 million units of personal protective equipment, testing kits and ventilators to over 150 countries and regions, including the United States and over 30 countries in Africa. Although this was not the largest COVID-mitigating financial donation by a wealthy donor, it was arguably the most impactful as Alibaba’s logistic muscle allowed the rapid delivery of life-saving face masks and ventilators to many countries that were outcompeted during the global jostle for life-saving equipment.^25^ On 16 March 2020, Jack Ma announced a donation of 100 000 masks, 20 000 test kits and 1000 sets of protective clothing and face shields to each of the 54 nations on the African continent.^26^ On 06 April 2020, an additional donation of medical supplies to all 54 countries of Africa was announced.^27^ This second donation included 500 ventilators, 200 000 sets of protective clothing and face shields, 2000 thermometers, one million swabs and extraction kits, and 500 000 gloves.

This brief article does not capture all the activities of all the partners that helped establish laboratory capacity for COVID-19 diagnosis in the early days of the pandemic in Africa. For example, many government and private partners, including the Ethiopian government, the Africa CDC, the United Nations World Food Programme, the WHO, and Ethiopian Airlines, helped distribute Jack Ma’s large donation. There were many other groups involved in the early days of the coronavirus laboratory response by countries in Africa, including Wellcome, the Department for International Development, the Mérieux Foundation, Oxford Nanopore Technologies, the United States CDC, and others.^20,28,29^

The denominating factor across the activities by all the players in this story was the speed with which they responded to build capacity. Having a pandemic nipping at one’s heels is a strong incentive to move fast and stay focused. Although the lightning-fast response by all partners to the COVID-19 pandemic described in this article may not be possible under normal operating conditions, it does demonstrate what is feasible and gives us something to aspire to in terms of speed and efficiency.

### Trusted institutions are crucial for moving Africa forward

Another lesson from this experience is that trusted institutions are the key to effective public health interventions. Much of the credit for the rapid establishment of testing capacity should go to the Africa CDC as the main coordinating body for external partners (the WHO, donors, etc.) and countries within Africa. But how was a three-year-old institution so effective at such a scale? The Africa CDC had obvious advantages such as the resources to hire and retain world-class staff, deep institutional connections to the WHO and the United States CDC, as well as the pressure of a pandemic. However, our opinion is that the Africa CDC was so effective because it is a trusted institution. Being a trusted institution does not mean that an institution is perfect or even efficient. Rather, a trusted institution has ‘policies and mechanisms that showcase their commitment to transparency, high standards, fiscal management, measurable results, and zero tolerance for corruption and mismanagement of funds’ (Brough R, April 2018, personal communication).

The establishment of COVID-19 testing capacity in Africa, while remarkable, was not an unmitigated success. African countries are still behind high-income countries in terms of the number of tests done per capita.^30^ Nevertheless, it was a remarkable achievement and provides a clear example of the positive impact of trusted institutions in public health.

## Recommendations

We need to build more local trusted institutions. Trusted institutions are characterised by predictability and transparency, which makes them more effective than other institutions because partners can engage without concerns that their donations may be diverted. More so, the influence of trusted institutions goes beyond donors and philanthropists as it also makes it easier for the recipients to engage. When invited by the Africa CDC for training or capacity building, countries are much more likely to respond positively because they know that such offers are usually without any hidden motives or expectations. Naturally, this begs the question, the answers to which are beyond the scope of this article but have been comprehensively addressed elsewhere^31,32^: how do we build trusted institutions?
